# The clinical spectrum and immunopathological mechanisms underlying ZIKV-induced neurological manifestations

**DOI:** 10.1371/journal.pntd.0009575

**Published:** 2021-08-05

**Authors:** Igor Salerno Filgueiras, Amanda Torrentes de Carvalho, Daniela Prado Cunha, Dennyson Leandro Mathias da Fonseca, Nadia El Khawanky, Paula Paccielli Freire, Gustavo Cabral-Miranda, Lena F. Schimke, Niels Olsen Saraiva Camara, Hans D. Ochs, Jean Pierre Schatzmann Peron, Otávio Cabral-Marques, Zilton Farias Meira de Vasconcelos

**Affiliations:** 1 Department of Immunology, Institute of Biomedical Sciences of University of São Paulo, São Paulo, Brazil; 2 Department of Immunobiology, Institute of Biology of Federal University of Fluminense, Niterói, Rio de Janeiro, Brazil; 3 Department of Clinical Research, Instituto Fernandes Figueira, Fiocruz, Rio de Janeiro, Brazil; 4 Department of Clinical Analyses and Toxicology, School of Pharmaceutical Sciences, University of São Paulo, São Paulo, Brazil; 5 Department of Hematology and Oncology, Faculty of Medicine, the University of Freiburg, Freiburg, Germany; 6 Department of Pediatrics, University of Washington School of Medicine and Seattle Children’s Research Institute, Seattle, Washington, United States of America; 7 Network of Immunity in Infection, Malignancy, and Autoimmunity (NIIMA), Universal Scientific Education and Research Network (USERN), São Paulo, Brazil; University of Texas Medical Branch, UNITED STATES

## Abstract

Since the 2015 to 2016 outbreak in America, Zika virus (ZIKV) infected almost 900,000 patients. This international public health emergency was mainly associated with a significant increase in the number of newborns with congenital microcephaly and abnormal neurologic development, known as congenital Zika syndrome (CZS). Furthermore, Guillain–Barré syndrome (GBS), a neuroimmune disorder of adults, has also been associated with ZIKV infection. Currently, the number of ZIKV-infected patients has decreased, and most of the cases recently reported present as a mild and self-limiting febrile illness. However, based on its natural history of a typical example of reemerging pathogen and the lack of specific therapeutic options against ZIKV infection, new outbreaks can occur worldwide, demanding the attention of researchers and government authorities. Here, we discuss the clinical spectrum and immunopathological mechanisms underlying ZIKV-induced neurological manifestations. Several studies have confirmed the tropism of ZIKV for neural progenitor stem cells by demonstrating the presence of ZIKV in the central nervous system (CNS) during fetal development, eliciting a deleterious inflammatory response that compromises neurogenesis and brain formation. Of note, while the neuropathology of CZS can be due to a direct viral neuropathic effect, adults may develop neuroimmune manifestations such as GBS due to poorly understood mechanisms. Antiganglioside autoantibodies have been detected in multiple patients with ZIKV infection–associated GBS, suggesting a molecular mimicry. However, further additional immunopathological mechanisms remain to be uncovered, paving the way for new therapeutic strategies.

## Introduction

Arthropod-borne viruses or arboviruses are responsible for many important infectious diseases worldwide [1]. Due to many aspects of modern society, such as disorganized urbanization, excessive population growth, and increasing international mobility in the past few decades, arboviral diseases currently represent a serious global public health issue [1]. In this context, the outbreak of Zika virus (ZIKV) infection that started in Brazil in 2015 was declared a state of emergency and global concern by the World Health Organization (WHO) on February 1 of the same year [1,2]. This was mainly driven by the exponential increase of newborns with microcephaly and adults with Guillain–Barré syndrome (GBS). Currently, there are approximately 4,000 cases of congenital Zika syndrome (CZS) in Brazil. The virus has spread to more than 94 countries, infecting as of today almost 900,000 people, confirming the relevance of arboviruses as a global threat [3].

ZIKV is mostly vectored by *Aedes aegypti* mosquitoes [4], followed by *Aedes albopictus* [5,6]. It is worth mentioning that it can also be found in human sperm up to 6 months after infection. Consequently, in September 2016, WHO further classified ZIKV infection as a sexually transmitted disease (STD) [7]. Furthermore, vertical transmission (mother-to-fetus and breastfeeding) and transmission by blood transfusions have been described, and the presence of ZIKV in tears was also reported [8–13]. Noteworthy, vertical transmission was also observed in vectors, as infected *Ae*. *aegypti* laid infected eggs [6]. Of note, scientists discovered that the *Ae*. *aegypti*, when exposed to ZIKV, chikungunya virus (CHIKV), and dengue virus (DENV), may transmit 1, 2, or even all 3 viruses simultaneously [14], resulting in viral coinfection and immune hyperresponsiveness [11].

Symptoms associated with acute phase ZIKV infection are headache, fever, conjunctivitis, myalgia, exanthem, and arthralgia [11], which may confound the initial diagnosis from other arboviruses such as DENV and CHIKV infections. Of note, approximately 80% of individuals infected with ZIKV do not develop any clinical manifestation [1], and only 0.3% to 0.5% of infected pregnant women have given birth to babies with microcephaly [15]. However, during the peak of the outbreak in Rio de Janeiro (2016), adverse neonatal outcomes reached 46% of the infected cases [16]. These facts indicate multifactorial influences in the outcome of ZIKV infection. In this context, Caires-Júnior and colleagues described that only one of dizygotic twins developed CZS [17]. This suggested that host factors (e.g., genetic background and epigenetics) also affect the outcome of ZIKV infection. On the other hand, viral mutations have been shown to be involved in pathogenicity and transmission [18]. In this regard, genetic and phylogenetic investigations indicate that distinct ZIKV lineages (e.g., those of West African, East African, and Asian origins) may affect infectivity, virulence, and clinical presentation. Thus, genetic aspects of the virus need to be considered in the etiopathogenesis of ZIKV infections and outcomes, which have been reviewed in detail elsewhere [19,20].

Many studies have confirmed the tropism of ZIKV for neural progenitor stem cells [21,22] and a causal relationship between ZIKV infection during fetal development and the occurrence of CZS [4,23,24]. The neural cell adhesion molecule (NCAM1) has been recently reported as the possible ZIKV receptor [25]. However, other entry receptors might be involved since AXL receptor tyrosine kinase (AXL) has been shown to mediate ZIKV entry in human glial cells [26]. For instance, it has been shown that the ZIKV genome interacts with Musachi-1 (MSH-1), an RNA-binding protein in the central nervous system (CNS). MSH-1 has an important function in orchestrating mRNA translation for proper neurodevelopment. ZIKV sequesters MSH-1 to promote its replication in the replisomes, impairing the translation of endogenous neurotrophic factors [27].

Notably, an association between ZIKV infection in adults and the development of autoimmune manifestations such as GBS has also been extensively reported [28]. However, the etiopathology remains not fully understood, thus making the investigation of ZIKV–host interactions an important research area to be explored. For instance, there is no specific therapy available for ZIKV, demanding the identification of novel immunopathological mechanisms to develop new therapeutic strategies. Here, we review the neuroimmunopathological mechanisms and disease outcomes associated with ZIKV infection.

## The clinical spectrum of congenital Zika syndrome

**Fig 1:**
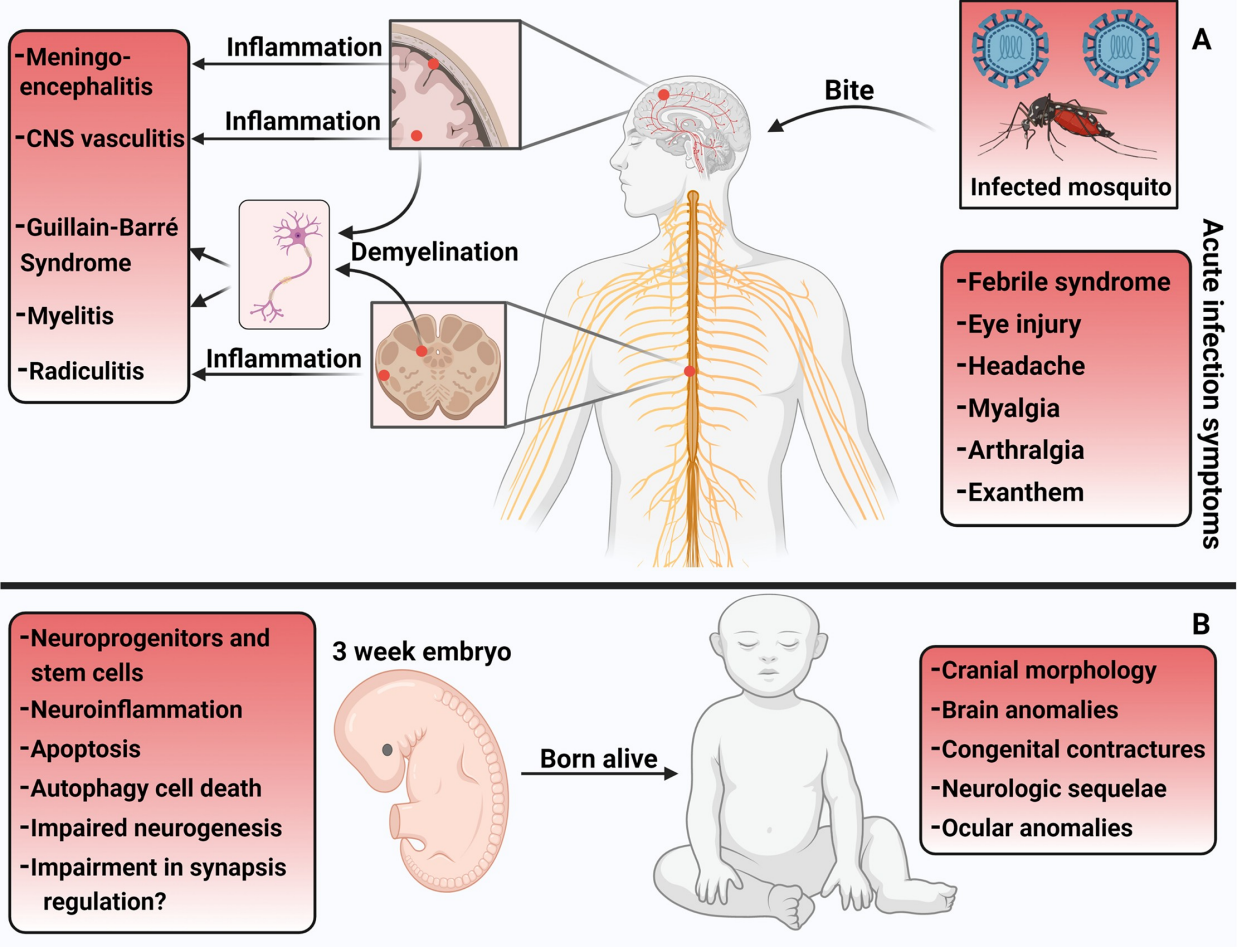
ZIKV-associated neurological manifestations. (**A**) Infected adults may be asymptomatic or present with febrile acute infection symptoms (right panel). The infection may also result in neurological complications, involving the CNS and/or PNS. (**B**) ZIKV infection during pregnancy can affect the fetus and result in abnormal nervous system development, impairing neurogenesis and leading to characteristic anomalies. Created with Biorender.com. CNS, central nervous system; PNS, peripheral nervous system; ZIKV, Zika virus.

The epidemic of CZS was first reported in Brazil in 2015. The many neurologic abnormalities of CZS may consist of abnormal cranial morphology such as overlapping cranial sutures, severe microcephaly, protruding occipital bone, neurologic impairment, and scalp ridges. Brain anomalies can also be present and include abnormal gyral patterns, thin cerebral cortices, larger amount of fluid spaces, calcification of subcortical regions, corpus callosum abnormalities, asymmetric and increased ventricles, lesser amount of white matter, and cerebellar hypoplasia. Ocular anomalies, if present, consist of macular pucker formation, retinal inflammation, focal pigmentary retinal mottling, and hypoplasia or atrophy of the optical nerve. Congenital contractures such as arthrogryposis and congenital talipes equinovarus have also been described. When neurologic sequelae occur, they may include early hypertonia, epilepsy, irritability, and symptoms of extrapyramidal involvement [16,29,30] (Fig 1).

## The immune response during pregnancy and the development of congenital Zika syndrome

During pregnancy, the female body employs homeostatic strategies to promote both immune regulation and tolerance while promoting immune surveillance and defense. These immunological processes support embryo development and prevent maternal–fetal infections to avoid placental dysfunction and intrauterine growth restriction [31]. The placenta provides a physical barrier interfacing the maternal and fetal blood circulation and is also essential for waste, gas, and nutrient exchange. The placenta is an immunologically active barrier to secure implantation and to restrict pathogen invasion [32]. The development of the fetus leads to an expansion of maternal peripheral blood mononuclear leukocytes and their recruitment into the placenta, promoting a tolerogenic environment [33]. In addition, extrinsic and intrinsic factors such as nutritional status, infections, stress, and obesity of a pregnant woman can influence her immune response and promote disorders associated with fetal neurodevelopment [34].

ZIKV can overcome the maternal–fetal immune physiological barrier by directly inducing cytopathic cell death and indirectly by tissue damage caused by a local exacerbated inflammatory response [22,35,36]. For instance, a detrimental role of type I interferons (IFNs) in pregnancy has been suggested when mice were inoculated with ZIKV at a gestational age corresponding to the mid and late first trimester in humans. This indicates a gestation stage–dependent ZIKV vertical transmission. By breeding homozygous type I IFN receptor (IFN-α/β receptor (IFNAR)) knockout (−/−) mothers with heterozygous male mice (IFNAR +/−), Yockey and colleagues showed that IFN-β produced in the placenta of heterozygous (IFNAR +/−) litters induced a more pronounced tissue damage and increased viral loads than in homozygous deficient ones (IFNAR−/−) [37]. These findings suggest that the signaling events triggered by IFN-β lead to abortion and growth restriction during ZIKV infection. This observation highlights the complex interplay between host and pathogen during ZIKV infection.

Moreover, new immunopathological mechanisms suspected to be involved in CZS await further investigation. For instance, ZIKV neurotoxicity could impair neurogenesis through a direct cytopathic effect on developing neurons by recruiting leukocytes and activating astrocytes and microglia. This may lead to subsequent congenital abnormalities and/or abortion. Of note, during brain inflammatory responses, astrocytes and microglia express inducible nitric oxide synthase (iNOS) [38]. Diop and colleagues have demonstrated that during initial hours of in vitro infection of microglia (CHME-5 cell line) with ZIKV, there is an up-regulation of chemokine receptors transcripts involved in leukocyte migration and synapse regulation as well as increase of iNOS and pro-inflammatory molecules such as tumor necrosis factor alpha (TNF-α), interleukin 1 beta (IL-1β), and IL-6 [39]. Nitric oxide (NO) is a gaseous bioactive compound that exerts protective and regulatory function on different cell types and influences the vascular smooth muscle tone. However, NO can present both anti- and proapoptotic properties, depending on its concentration and source. At low concentrations and when derived from endothelial and neuronal isoforms of NO synthase (eNOS and nNOS), NO normally has protective effects. On the other hand, at higher concentration levels and derived from iNOS, NO is more likely to induce cell death [40]. Therefore, low levels of NO promote the destruction of microorganisms and tumor cells, but at high concentrations and for long term, it induces apoptosis of neurons, genotoxic species, and neurodegenerative disorders caused by an S-nitrosylation–dependent pathway [41].

## Neuroimmune disorders associated with ZIKV infections

In addition to CZS, a variety of neurological manifestations affecting both CNS and peripheral nervous system (PNS) of adults have been reported in patients infected with ZIKV, such as GBS, CNS vasculitis, radiculitis, myelitis, meningoencephalitis, or a combination of these complications [28,42] (Fig 1). While CZS is the direct result of the neuropathological effects of the virus [22], these neuroimmune manifestations may occur due to immune dysregulation and autoimmunity triggered after convalescence of ZIKV infection, at least in some patients [43]. This hypothesis is based on postmortem examination of some infected adults in whom ZIKV viral RNA or antigen were undetectable in the PNS and CNS of patients who had developed GBS.

### Guillain–Barré syndrome

**Fig 2:**
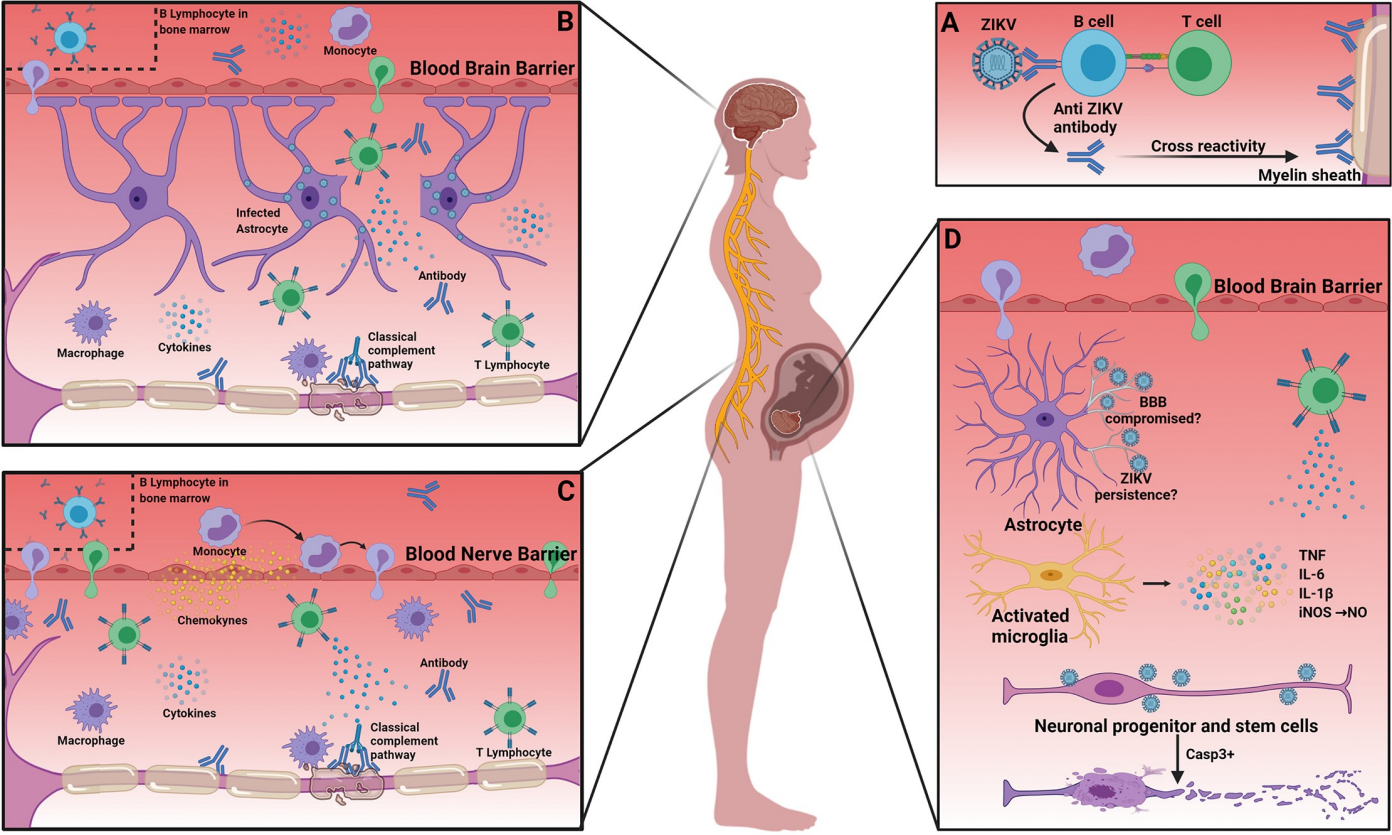
Neuroimmunological mechanisms involved in ZIKV infection. (**A**) Molecular mimicry between ZIKV and gangliosides. (**B**) Infected astrocytes mediate the activation and recruitment of immune cells into the CNS. (**B, C**) Antibodies against ZIKV cross-react with neurons, eliciting an autoimmune response that results in demyelination. (**D**) During fetal development, local infection activates microglia, promoting the production of cytokines. Loss of BBB integrity occurs due to infection of astrocytes, which allows immune cell recruitment, contributing to pathological neuroinflammation. Neural stem cell infection results in autophagy and possibly other unknown immunopathological mechanisms. The impaired neurogenesis results in congenital abnormalities. Created with Biorender.com. BBB, blood–brain barrier; CNS, central nervous system; IL-1β, interleukin 1 beta; IL-6, interleukin 6; iNOS, inducible nitric oxide synthase; NO, nitric oxide; TNF, tumor necrosis factor; ZIKV, Zika virus.

GBS is an autoimmune disease characterized by progressive bilateral weakness and loss of deep tendon reflexes due to peripheral nerve damage [28,43]. The etiopathogenesis of GBS following ZIKV infection may involve molecular mimicry between glycolipids and some ZIKV structural molecules, thus leading to an autoimmune response [44]. A recent systematic review and meta-analysis by Leonhard and colleagues characterized the clinical phenotype of ZIKV-associated GBS as a general sensorimotor demyelinating syndrome with frequent facial paralysis [45]. The authors observed that the time between the development of infectious symptoms and neurologic manifestations was approximately 1 week, and ZIKV viral RNA could be detected in the cerebrospinal fluid (CSF) through reverse transcription polymerase chain reaction (RT-PCR) in only 10 out of 244 cases. While this observation suggested that the outcome of ZIKV infection may involve host self-reactivity, the authors were not able to exclude the possibility that the ZIKV may directly trigger GBS while hiding in CNS or PNS compartments. Of note, some of these individuals showed demyelination associated with inflammation and mononuclear lymphocytic infiltration [28], while others were found to have autoantibodies [46] (Fig 2). Beyond, there is evidence that the development of GBS not associated with ZIKV infection involves the activation of the classical complement pathway, disrupting myelin sheath, nodes of Ranvier, and other membranes of the nervous system [47]. However, the role of the complement system in ZIKV-induced GBS remains to be investigated.

### Encephalitis

A recent study performed in Colombia, where a high number of pediatric ZIKV infections occurred, identified a total of 6 encephalitis cases in children [48]. The symptoms diminished faster than those of encephalitis caused by other infectious agents. Lymphocytosis of the CSF was present in all cases, and higher cytokine levels were found in the CSF of 1 patient when compared with plasma levels, suggesting local inflammation [48].

A single patient with preexisting multiple sclerosis (MS), who developed acute disseminated encephalomyelitis following ZIKV infection, indicates the possibility that this virus may exacerbate MS symptoms [49]. The patient was found to have ZIKV envelope protein in the brain tissue, indicating the presence of the virus, possibly due to disruption of the blood–brain barrier (BBB) directly related to MS. These data suggest an association between neurologic complications due to ZIKV and an existing immune dysregulation background. However, similar cases are necessary to strengthen this hypothesis. These events raise the question whether ZIKV primary infections in the CNS may lead to severe neuropathological complications in patients with a preexisting MS condition.

### Neuromyelitis optica spectrum disorder

Another possible outcome of ZIKV infection is the occurrence of neuromyelitis optica spectrum disorder (NMOSD), recently characterized in a single patient [50]. NMOSD is a severe and debilitating condition that mostly affects the spinal cord. The development of NMOSD not associated with ZIKV infection involves antibodies, mainly immunoglobulin G (IgG), against a water channel called aquaporin-4 (Aqua-4) that is mostly expressed by astrocytes. This leads to complement activation as well as antibody-dependent cellular cytotoxicity (ADCC), resulting in extensive damage, as shown by MRI [51,52]. Initially, the patient with ZIKV infection presented with a positive RT-PCR for the virus in the CSF. Later in the course of the disease, he developed tactile and temperature allodynia of both arms. However, the immunopathological mechanism underlying the development of NMOSD remains to be uncovered. For instance, whether there is a molecular mimicry between ZIKV antigens and Aqua-4 has not been investigated.

## The immune response to ZIKV and its tropism for the nervous system

Blood mononuclear cells such as antigen-presenting cells (APCs; e.g., monocytes and dendritic cells) are the most frequently infected leukocyte subpopulations by ZIKV [53]. The recognition of pathogen-associated molecular patterns (PAMPs) of ZIKV (e.g., RNA) by host pathogen recognition receptors (PRRs) such as the Toll-like receptor 3 (TLR3) is followed by the up-regulation of pro-inflammatory molecules [54]. For example, the recognition of ZIKV by APCs induces the production of pro-inflammatory mediators and microbicidal mechanisms, such as production of IFNα/β, TNF-α, IL-1β, and NO. These innate immunity events are essential for the activation of T and B lymphocytes, responsible for the adaptive immune response [55], and, consequently, viral control and elimination [56].

In general, APCs present in human blood and epidermis seem to be the main route of distribution of the virus to other host tissues [56]. However, ZIKV also infects several other cell types such as skin epithelial cells [57], trophoblasts [58], neuronal progenitors, and stem cells [4,22]. During replication, many flaviviruses induce the rearrangement of the endoplasmic reticulum (ER) membrane to support viral production. This process triggers ER stress that results in an active unfolded protein response and autophagy [59], which is a constitutive process of antigen presentation, but is also potentiated during stress, such as nutrient deprivation. Most cells catabolize proteins to generate energy by carrying and degrading damaged organelles and cytosolic proteins in lysosomes or more complex structures generated by the fusion of these with autophagosomes. If autophagy is up-regulated and persists, cells may die [60]. In this context, ZIKV infection of human fetal neural stem cells may impair neurogenesis by aberrant activation of autophagy, i.e., the nonstructural viral proteins NS4A and NS4B synergistically induce cellular dysregulation by suppressing the PI3K-Akt-mTOR pathway [61], which is essential for brain development and autophagy regulation [62,63].

It has been shown that ZIKV colocalizes with autophagosomes [57]. Souza and colleagues developed a biological system of induced neural differentiation obtained by reprogramming human skin fibroblasts. The in vitro infection of neural stem and progenitor cells with ZIKV results in the depletion of progenitors and disruption of neural differentiation, as demonstrated by transmission electron microscopy and confocal microscopy [64]. The authors showed impaired cell proliferation and down-regulation of caspase-dependent apoptotic cell death. They also confirmed the occurrence of autophagy by the presence of numerous autophagosomes in the perinuclear region of ZIKV-infected cells.

### Type I IFNs and signaling pathways involved in the immune response to ZIKV

Among others, type I IFNs trigger the activation of the signal transducer and activator of transcription 1 (STAT1) and STAT2, which play a key role in the antiviral immune response [65]. They induce a state of viral resistance in host cells by activating enzymes such as 2′-5′-oligoadenylate synthetase 1 (OAS1) or ribonuclease L (RNAse L) that catalyze viral RNA degradation [66]. Type I IFNs are also responsible for the up-regulation of class I major histocompatibility complex (MHC) and costimulatory molecules (CD80, CD86, and CD40), potentializing antigen presentation to T helper (CD4+) and cytotoxic (CD8+) lymphocytes [67]. These IFNs also increase cytolytic action and proliferation of natural killer (NK) cells through the production of cytokines such as IL-15 [68]. Jurado and colleagues [69] demonstrated that transgenic mice lacking type I IFNs have increased viremia in the CNS when infected by ZIKV, mostly due to the antagonistic effect of the ZIKV nonstructural protein 5 (NS5) on STAT1 and STAT2 phosphorylation induced by type I IFNs [70].

In addition, in vitro infection of human-induced neural progenitor cells (hiNPCs) by a ZIKV Brazilian strain showed a transcriptional profile related to inflammation, IFN response, cell death, and growth [71]. Lima and colleagues confirmed these data at the protein level by measuring soluble cytokines and chemokines in hiNPCs supernatants using a multiplex assay [71]. The levels of type I IFNs and of chemokines and cytokines associated with effector leukocyte recruitment and pro-inflammatory mechanisms were also significantly higher in CSF samples of CZS infants [71]. This raises the possibility that ZIKV affects infants’ brains, triggering a local pathological inflammation that compromises neurogenesis and brain development.

### ZIKV and immune evasion strategies

ZIKV avoids host immune response by a number of mechanisms, favoring viral replication and vertical transmission. Studies performed both with human cells and animal models clearly demonstrated the importance of type I and III IFNs in the prevention of ZIKV infection [24,72]. Type I IFNs activate cells expressing the IFNAR. This event triggers STAT1, STAT2, and IFN regulatory factor 9 (IRF-9) to translocate to the nucleus and induce the transcription of multiple antiviral proteins called IFN-stimulated genes (ISGs), which effectively block viral replication and viral particle assemblage. Interestingly, the ZIKV NS5 protein targets human STAT2 inducing its degradation, abolishing type I IFNs responses [70]. However, this phenomenon is not observed in mice. For this reason, mice are highly resistant to ZIKV infection compared to humans, and transgenic or IFN knockout models are required to further investigate the host–pathogen relationship [24,73].

It has also recently been shown that ZIKV triggers the production of kynurenine (Kyn), which activates its receptor called aryl hydrocarbon receptor (Ahr). This receptor is capable of suppressing not only type I IFNS, but also inhibits the effect of the promyelocytic protein leukemia (PML) protein, which limits ZIKV replication. The use of Ahr antagonists in a murine experimental model of vertical transmission abrogated kyn-induced suppression and led to a better fetal outcome [74].

## A possible implication of pyroptosis and inflammasome activation in neurological disorders associated with ZIKV infection

Pyroptosis is a type of programmed cell death triggered by the stress of extracellular or intracellular homeostasis [75]. Morphologic alterations associated with pyroptosis are a unique form of chromatin condensation that differs from apoptosis and plasma membrane permeabilization. Pyroptosis utilizes caspase-1–dependent and caspase-1–independent mechanisms. During dengue infection, viral RNA is recognized by innate receptors [76]. Cytoplasmic nucleotide-binding oligomerization domain (NOD)-like receptors activate signaling pathways that subsequently culminate in the activation of a multiprotein complex called inflammasome. Among the components that make up this multiprotein unit, the cellular protease caspase-1 is of relevance [77]. With the activation of inflammasomes, pro-caspase-1 is cleaved into caspase-1, which then cleaves pro-IL-1β and pro-IL-18 as well as gasdermin D (GSDMD), which are released to the extracellular milieu [78]. IL-1β is a pyrogenic cytokine that mediates fever, immune cell migration, BBB disruption, adaptive immune activation, and several other functions. IL-18 induces IFN-γ production, which is important to activate effector T cells and NK cells. In turn, GSDMD is known to form pores that are necessary for the rapid release of IL-1β [79,80]. A recent study demonstrated that inflammasome responses are associated with human glioblastoma cell line activation when infected with ZIKV. This infection was linked with increased oxidative stress and pyroptosis, contributing to inflammation and neurological dysfunction [81].

## The protective role of microglia against neurological damage caused by ZIKV

Microglia are resident macrophages located in the CNS. They are a main component of the local immune response, eliminating apoptotic cells and playing an essential role in brain development, synaptic pruning, memory, and neuronal recycling [35]. Limonta and colleagues demonstrated that primary human fetal astrocytes (HFAs) that promote neuron support and nutrition and participate in the BBB maintenance can become a reservoir for ZIKV, shedding virus for at least 1 month post-in vitro infection [82]. ZIKV infects microglia progenitors that derive from the yolk sac (first site of hematopoiesis in both mice and humans), and then, when mature, microglia carrying ZIKV invade the fetal brain [82]. This provides an explanation on how ZIKV reaches the brain of the fetus.

Moreover, many PAMPs and damage-associated molecular patterns (DAMPs) are associated with brain damage and can trigger microglia activation. Fekete and colleagues hypothesized that microglia sense damage of individual cells before irreversible neuronal injury, recruiting more phagocytic resident cells to the compromised neurons after virus infection via purinergic receptors, promoting phagocytosis, and restraining virus dissemination [83]. The authors also demonstrated that ATP delivered by infected neurons exerts chemotactic function, recruiting more precursors of phagocytic cells into the site of brain infection [83].

## Conclusions

Despite the advances in understanding the immunopathology of the neurological disorders associated with ZIKV infections, several underlying mechanisms remain poorly understood. One important aspect is to determine the main routes and biological processes of ZIKV infection in fetuses and adults and whether they overlap. In addition, extensive research is necessary to find therapeutic targets to avoid uncontrolled ZIKV-induced neuroinflammation and BBB damage. This is important to prevent the resulting neuropathology due to cell death, dysregulated cell cycle–related pathways, and local immune dysregulation [71,84].

Although the role of type I IFNs and their associated signaling pathways involved in the immune response to ZIKV have been extensively investigated, other protective host factors remain to be uncovered. In addition, immunopathological mechanisms that are poorly understood in humans have been investigated in detail in ZIKV-infected mice, including damage of the BBB by astrocytes resulting in a significant infiltration of T lymphocytes into the CNS. This event destroys neurons, causing considerable damage to the brain due to dysregulation of antiviral activity and cytotoxicity resulting in paralysis. This finding supports the role of adaptive immunity in the neurological manifestations that occur in ZIKV-infected patients [69,85]. However, the precise mechanisms resulting in immune dysregulation that lead to ZIKV-induced autoimmunity remain unclear. Thus, in the absence of approved specific anti-ZIKV therapy or vaccines, a better understanding of the mechanisms that are involved in susceptibility to ZIKV infection and its pathophysiology will be essential for developing effective therapies, reducing morbidity and mortality of newborns and adults due to ZIKV infections.

**Key Learning Points**

- The development of severe outcomes due to Zika virus (ZIKV) infections relies on the host’s immune response.
- Immunoglobulin G (IgG) and IgM autoantibodies are associated with the development of Guillain–Barré syndrome (GBS) in patients infected by ZIKV.
- The inflammatory response compromises neurogenesis and brain development during ZIKV infections.
- There is an association between neurological complications due to ZIKV and an existing autoimmune dysregulation background.

**Top Five Papers**

1. da Silva IRF, Frontera JA, Bispo de Filippis AM, Nascimento OJM do. Neurologic Complications Associated With the Zika Virus in Brazilian Adults. JAMA Neurol. 2017;74:1190. doi: 10.1001/jamaneurol.2017.1703
2. Caires-Júnior LC, Goulart E, Melo US, Araujo BSH, Alvizi L, Soares-Schanoski A, et al. Discordant congenital Zika syndrome twins show differential in vitro viral susceptibility of neural progenitor cells. Nat Commun. 2018;9. doi: 10.1038/s41467-017-02790-9
3. Srivastava M, Zhang Y, Chen J, Sirohi D, Miller A, Zhang Y, et al. Chemical proteomics tracks virus entry and uncovers NCAM1 as Zika virus receptor. Nat Commun [internet]. 2020 Dec 4;11(1):3896. Available from: http://www.nature.com/articles/s41467-020-17638-y.
4. Rivera-Correa J, de Siqueira IC, Mota S, do Rosário MS, Pereira de Jesus PA, Alcantara LCJ, et al. Anti-ganglioside antibodies in patients with Zika virus infection-associated Guillain-Barré Syndrome in Brazil. Christofferson RC, editor. PLoS Negl Trop Dis. 2019;13:e0007695. doi: 10.1371/journal.pntd.0007695
5. Giovannoni F, Bosch I, Polonio CM, Torti MF, Wheeler MA, Li Z, et al. AHR is a Zika virus host factor and a candidate target for antiviral therapy. Nat Neurosci. 2020;23:939–951. doi: 10.1038/s41593-020-0664-0
